# Prevalence, antibiotic resistance patterns, and virulence factors of *Staphylococcus aureus* isolates associated with bovine mastitis in northern Bangladesh

**DOI:** 10.1016/j.heliyon.2025.e42107

**Published:** 2025-01-21

**Authors:** Md. Mominul Islam, Md. Imran Hossain, Md. Sadequl Islam, Md. Golam Azam, Sajeda Sultana

**Affiliations:** a Department of Pathology and Parasitology, Hajee Mohammad Danesh Science and Technology University, Dinajur, 5200, Bangladesh; b Department of Pathology, Sher-e-Bangla Agricultural University, Sher-e-Bangla Nagar, Dhaka, 1207, Bangladesh; c Department of Anatomy and Histology, Hajee Mohammad Danesh Science and Technology University, Dinajur, 5200, Bangladesh

**Keywords:** *Staphylococcus aureus*, Bovine mastitis, Antibiotic resistance, Virulence factors

## Abstract

*Staphylococcus (S.) aureus* is a major cause of bovine mastitis and is notorious for its capacity to resist antibiotics, presenting substantial risks to both livestock and human health. The aim of this research was to assess the prevalence of *S. aureus* in bovine mastitis cases, as well as to examine their patterns of antimicrobial resistance and virulence genes contributing to mastitis in cattle. For this study, 120 milk samples were gathered from clinically mastitis affected cows across three districts in the northern part of Bangladesh. The detection and confirmation of *S. aureus* involved standard microbiological and biochemical techniques. The antibiotic sensitivity of the strains was evaluated using the disk diffusion method with a variety of antibiotics frequently used in veterinary settings. Furthermore, PCR was utilized to explore the presence of virulence genes linked to the pathogenicity of *S. aureus*. Findings revealed that out of the sampled cases, 56 strains of *S. aureus* were isolated, indicating a prevalence rate of 46.66 % in cases of clinical mastitis. The results revealed a diverse range of antibiotic resistance patterns among the isolates, with a notable prevalence of resistance to penicillin (100 %), ampicillin (85 %), amoxicillin (75 %), tetracycline (66 %), chlortetracycline (64 %), azithromycin (57 %), kanamycin (54 %), and gentamicin (50 %). 36 isolates out of 56 (64 %) were multidrug resistant in nature. Furthermore, virulence gene profiling identified the genes responsible for biofilm formation (*bap*), adhesion, inflammation and tissue damage (*seb*, *pvl*), and toxin production (*hla* and *hlb*), indicating the potential pathogenicity of the isolates. Notably, 12 isolates (21.42 %) harbored gene linked to methicillin resistance (*mecA*), raising concerns about the potential transmission of antimicrobial-resistant *S. aureus* strains from dairy cows to humans through the food chain. These findings underscore the critical importance of implementing stringent antimicrobial stewardship practices and surveillance measures in dairy farming to mitigate the dissemination of antibiotic resistance.

## 1 Introduction

Mastitis in cows is one of the most prevalent and economically significant diseases affecting the dairy industry worldwide. It leads to substantial economic losses by reducing milk production, necessitating the disposal of milk containing antibiotic residues, increasing treatment costs, and resulting in the culling of cows with chronic mastitis, as well as higher animal mortality rates [1]. Numerous microorganisms, including bacteria, viruses, and fungi, are responsible for mastitis in cows, with *Streptococcus* spp.*, Staphylococcus* spp.*, and E. coli* recognized as the major pathogens [2]. *S*. *aureus*, a Gram-positive bacterium, plays an important role in veterinary and human medicine due to its capacity to cause a broad spectrum of infections. In the context of dairy farming, it is notably recognized as a predominant etiology of bovine mastitis due to its high virulence factors [3,4] multidrug resistant strains [5] and diverse host pattern [6]. These virulence genes responsible for evading the host immune response, establish intramammary infection and at least in some extent also responsible for different clinical outcomes, extent and severity of infections, antibiotic responses to infection [7]. The treatment of cow affected with bovine mastitis includes use of wide range of antibiotics and improve animal husbandry of the farm.

The growing problem of antimicrobial resistance increasingly complexes the cure of mastitis, complicating the management of diseases caused by pathogens like *S. aureus*. Antibiotics are widely used for therapeutics, prophylactics and growth promoting purpose in low middle income country. Many antibiotics for example beta-lactums, macrolides, tetracycline etc. were used for treatment of bovine mastitis. However, addressing mastitis induced by *S. aureus* has grown more complex over time due to the rise of antibiotic-resistant microbes, particularly methicillin-resistant *Staphylococcus aureus* (MRSA) [8]. *S. aureus* shows resistant to common antimicrobials by preventing bacterial cell penetration forming exopolysaccharide layer [9]. Furthermore, acquired resistance can develop through genes located on plasmids or via transposons [10]. In *S. aureus*, methicillin resistance is linked to the presence of the *mecA* gene, which resides on the bacterial chromosome. This gene encodes penicillin-binding protein 2a (PBP2a), which significantly reduces the binding affinity of β-lactam antibiotics [11]. Additionally, MRSA strains isolated from mastitic milk have also been found to exhibit resistance to other classes of antibiotics, such as macrolides [12] and erythromycin [13].

*S. aureus* is well-known not only for its antibiotic resistance but also for its array of virulence factors. These include enterotoxins, hemolysins, leukocidins, and superantigens, which collectively contribute to the development of intramammary infections. These factors play a crucial role in helping the bacteria evade the host's immune defenses, further complicating infection management [7].The organism secretes four varieties of haemolysins: α, β, γ, and δ toxins, with α and β toxins being notably crucial for the pathogenicity of *S. aureus* [14]. Staphylococcal enterotoxins remain biologically active in the gastrointestinal tract following ingestion and interact with specific emetic receptors. Moreover, they become resistance to heat treatment and may maintain their biological potency in different circumstances [15]. Moreover, the development of biofilms, which are intricate assemblies of bacterial cells encased within a matrix they produce themselves, plays an important role in the antibiotic resistance of MRSA. These biofilms shield the bacteria from both the immune response and antibiotic treatments, greatly enhancing their resistance to therapy. The capability of MRSA to generate biofilms is controlled by various genes and signaling mechanisms, including the *icaABCD* operon, accessory gene regulatory system (*agr*) and biofilm associated protein (*bap*) [16].

Although there has been considerable research focused on the antibiotic resistance patterns and the virulence factors of *S. aureus* strains obtained from mastitis milk in other parts of world, comprehensive investigations of these strains in cases of mastitis, particularly examining their antibiotic resistance and virulence profiles in Bangladesh, are sparse [[17], [18], [19]]. This research investigates the occurrence of particular virulence factors in *S. aureus* strains, especially those that encode for enzymes and toxins which enhance the pathogen's capability to initiate and sustain infections. Through molecular characterization of virulence genes and antimicrobial susceptibility testing against clinically relevant antibiotics, this study aims to elucidate the interplay of antimicrobial resistance and virulence in *S. aureus* strains associated with mastitis.

## 2 Materials and methods

### 2.1 Experimental area

**Fig. 1:**
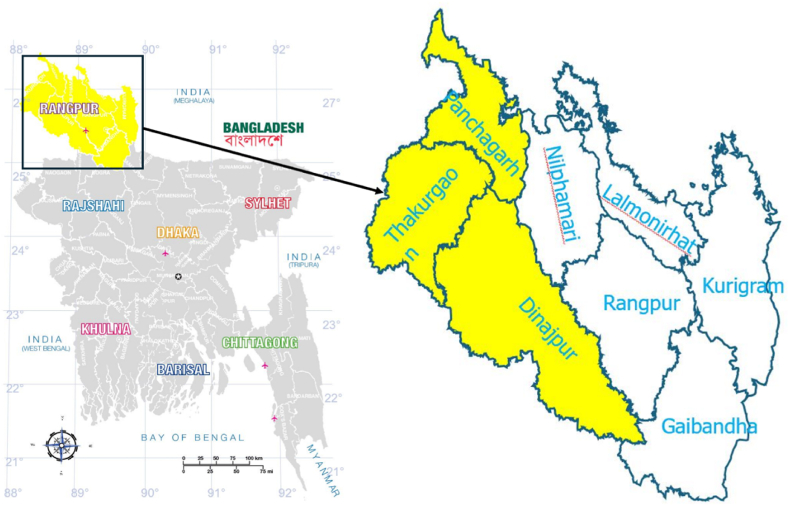
The study was conducted in the areas of Thakurgoan, Dinajpur and Nilphamari district (yellow color) of Bangladesh.

This study was performed in the Rangpur division, specifically within the northern districts of Thakurgaon (26.0330°N 88.4610°E), Dinajpur (25.6217°N 88.6355°E), and Nilphamari (26.3330°N 88.5500°E), in Bangladesh (Fig. 1). A collection of 120 milk samples were received from 120 cows displaying characteristics signs of bovine mastitis in the aforementioned three districts. These samples were collected from small-scale dairy farms (10–20 cows), and individual producers in 12 regions of the three districts during July 2022 to June 2023.

### 2.2 Sample collection

While collecting milk samples from clinically mastitis affected cows, the entire udder and teats of the cows underwent washing and drying procedures. Particular attention was paid to preventing contamination from the milker's hand. Clinical mastitis was confirmed by the veterinarian based on the abnormal udder and milk characteristics. Before collection of milk samples, no treatment were given to animal. Approximately 10 ml of milk was then collected in falcon tubes after discarding the initial stream from all quarters and mixed them properly. Then, the collected samples were transferred to the Microbiology lab for further processing.

Milk samples were stored only if they were collected just before the weekend. In such cases, samples were preserved at 4 °C and subsequently cultured on standard bacteriological media. We followed established guidelines for milk sample storage to ensure microbiological quality was maintained, and all samples were processed within a 24–36 h window.

### 2.3 Isolating and identifying bacteria

A milk sample was applied onto 5 % Sheep Blood Agar plates (HiMedia, India). These plates were subsequently kept in an incubator at 37 °C and inspected for growth following a 24-h period. The identification of colonies was performed based on their reaction to Gram's stain, colony features, and hemolytic behavior on blood agar. The colonies were moved to Mannitol Salt Agar (MSA) (HiMedia, India) and then kept at 37 °C. Afterward, colonies grown on MSA were transferred to nutritional media (HiMedia, India) to facilitate further growth. Coagulase, catalase, oxidase, and DNase tests were conducted, along with monitoring growth on MSA, following the methods outlined by Jahan et al. [19].

### 2.4 Susceptibility testing of *S. aureus* to antimicrobials

To assess the sensitivity of *S. aureus* to antibiotics, the disc diffusion technique was employed (Hoque et al., 2022). Oxacillin (30 μg), Cefoxitin (30 μg), Penicillin (30 μg), Ampicillin (30 μg), Amoxicillin (30 μg), Chlortetracycline (30 μg), Gentamycin (30 μg), Azithromycin (30 μg), Ciprofloxacin (30 μg), Tetracycline (30 μg) and Vancomycin (30 μg) were used for testing the isolated strains (Oxoid, England). The procedure was carried out on Muller Hinton agar plates (HiMedia, India) followed by the incubation of the plates at 37 °C for 24 h, adhering to the guidelines provided by the Clinical Laboratory Standards Institute for veterinary pathogen (VET08) [20].

### 2.5 Biofilm formation detection

To evaluate biofilm formation in all *S. aureus* isolates, the Congo red agar (CRA) method was employed. The CRA medium was formulated by mixing brain heart infusion broth (HiMedia, India), sucrose, agar (HiMedia, India), and a Congo red indicator. Following preparation, the *S. aureus* isolates were inoculated onto CRA plates and incubated at 37 °C for 24 h. Biofilm formation was indicated by the presence of black colonies with a dry, crystalline texture, whereas non-biofilm-forming isolates produced red or pink crystalline colonies.

### 2.6 DNA extraction and molecular detection of *S. aureus* and its virulence genes

DNA extraction from fresh *S. aureus* colonies was performed using the QIAamp DNA extraction kit (Qiagen, Germany), adhering to the manufacturer's protocol. Subsequent PCR analysis was conducted to identify *S. aureus* and its virulence genes, specifically targeting 23S rRNA, *mecA*, *sea*, *seb*, *sec*, *hla*, *hlb*, *pvl*, and *bap*. The sequences of the primers were listed in Table 1. Each PCR reaction consisted of 12.5 μL of 2 × Green Master Mix (Promega, USA), 5 μL of DNA template, 1 μL of each primer, and nuclease-free water to achieve a final volume of 25 μL. The PCR cycling conditions included 95 °C for 3 min as initial denaturation, then denaturation at 95 °C for 30s, annealing temperature was mentioned in table for each primer set, 72 °C for 45s for elongation, and a final extension step at 72 °C for 5 min. Amplicons were then visualized by electrophoresis on 2 % agarose gels prepared in 0.5 × Tris-borate-EDTA (TBE) buffer.

**Table 1:** Primer sequence of specific genes with their product size.

Genes	Primer sequences	Product size	Annealing temp	cycles	References
*23s rRNA*	F-GGA CGA CAT TAG ACG AAT CA	1318	60	35	(21)
	R-CGG GCA CCT ATT TTC TAT CT				
*coa*	**F-**ACCACAAGGTACTGAATCAACG	750	55 for 1 min	30	(22)
	**R**-TGCTTTCGATTGTTCGATGC				
*mecA*	F-AAAATCGATGGTAAAGGTTGGC	533	53	35	(23)
	R-AGTTCTGGAGTACCGGATTTGC				
*Sea*	**F-** GCAGGGAACAGCTTTAGGC	521	55 for 1 min	30	(24)
	**R-** GTTCTGTAGAAGTATGAAACACG				
*Seb*	**F-** ACATGTAATTTTGATATTCGCACTG	667	55 for 1 min	30	(24)
	**R-** TGCAGGCATCATGTCATACCA				
*sec*	**F-** CTTGTATGTATGGAGGAATAACAA	284	55 for 1 min	30	(24)
	**R-** TGCAGGCATCATATCATACCA				
*hlb*	F- GCCAAAGCCGAATCTAAG	840	55 for 1 min	30	(25)
	**R-** GCGATATACATCCCATGGC				
*bap*	F-CCCTATATCGAAGGTGTAGAATTGCAC	971	60	35	(26)
	R-GCTGTTGAAGTTAATACTGTACCTGC				
*pvl*	F-ATCATTAGGTAAAATGTCTGGACATGATCCA	433	50 °C for 45 s	35	(27)
	R- GCATCAAGTGTATTGGATAGCAAAAGC				

## 3 Results

### 3.1 The prevalence of *S. aureus* in mastitis affected cows

In this investigation, a total of 56 *S. aureus* strains were isolated and characterized from 120 samples of mastitis-infected milk. The identification of *S. aureus* was validated through assessment of cultural and biochemical traits. The strains exhibited mannitol fermentation, yielding colonies with a yellow hue on Mannitol Salt Agar. Additionally, catalase and coagulase assays were conducted, resulting in formation of bubbles and clot, respectively. The overall prevalence of *S. aureus* from mastitis case was determined as 46.66 % (Table 2).

**Table 2:** Prevalence of *Staphylococcus aureus* isolated from bovine clinical mastitis.

Regions	No. of samples	No. of positive samples	Prevalence (%)
Dinajpur	52	22	42.3
Nilphamari	42	16	38.09
Thakurgaon	26	18	69.23
Total	120	56	46.66

Highest prevalence of *S. aureus* was found in Thakurgoan (69.23 %), followed by Dinajpur (42.3 %) and Nilphamari (38 %), respectively (Table 2).

### 3.2 Antibiotic sensitivity pattern of isolated *S. aureus*

The resistance patterns of *S. aureus* isolates from samples of clinical mastitis were outlined in Table 4 S *aureus* showed resistance to antibiotics like penicillin (100 %), ampicillin (85 %), amoxicillin (75 %), tetracycline (66 %), chlortetracycline (64 %) azithromycin (57 %), Kanamycin (54 %),and gentamicin (50 %).The isolates exhibited susceptibility to certain antibiotics, including vancomycin (86 %), ciprofloxacin (70 %), cefoxitin (70 %), and oxacillin (68 %) (Table 3).

**Table 3:** Antibiotic sensitivity profiles of isolated *S. aureus*

Antimicrobial agents (ug/disc)	No. of isolates (%)		
	Resistant	Intermediate	Sensitive
**Amoxicillin (AMX-30)**	30 (54)	12 (22)	14 (25)
**Ciprofloxacin (CIP-5)**	8 (15)	7 (14)	41 (71)
**Kanamycin (K-30)**	12 (22)	18 (32)	26 (56)
**Gentamicin (Gen-10)**	14 (25)	14 (25)	28 (50)
**Oxacillin(OX-30)**	10 (18)	12 (22)	34 (68)
**Cefoxitin (CX-30)**	8 (15)	8 (15)	40 (70)
**Vancomycin (VA-30)**	2 (4)	6 (11)	48 (86)
**Chlortetracycline (CTR-30)**	25 (45)	9 (16)	22 (39)
**Azithromycin (AZM-30)**	14 (25)	18 (32)	24 (43)
**Ampicillin (AMP-25)**	32 (58)	15 (27)	9 (16)
**Penicillin (Pen-10)**	52 (93)	4 (7)	0 (0)
**Tetracycline (Tet-30)**	28 (50)	9 (16)	19 (34)

**Table 4:** Resistance to multiple drugs in isolates from clinical mastitis.

No. of pattern	Antibiotic resistance patterns	No. of antibiotics	No. of isolates
1	PEN, AMX, AMP, AZM, CTR, CX, GEN, OX, K	9	2
2	PEN, AMX, AMP, CIP, V	5	6
3	PEN, AMP, AZM, CTR, OX, K	6	4
4	PEN, AMX, CTR, V	4	2
5	PEN, AMP, AZM, GEN, CIP, K, OX	7	10
6	PEN, AMX, AMP, CTR, CX, OX	6	4
7	PEN, AMX, AMP, AZM, CTR, CX, GEN, K	8	8
8	PEN, AMX, AMP	3	20

Bacteria are classified as multidrug-resistant (MDR) if they show resistance to at least one antibiotic from three or more classes in in vitro susceptibility testing [28]. The multidrug resistance pattern of isolated *S. aureus* were shown in Table 4. Out of 56 isolates of *S. aureus*, 36 exhibited multidrug resistance (MDR). There were a total of 8 distinct resistance patterns observed, with 7 of them being characterized by MDR. The highest number of MDR *S. aureus* isolates (10/56) was found in the resistance pattern 5 (PEN, AMP, AZM, GEN, CIP, K, OX). In addition, 2 isolates of the pattern one showed resistance to 9 antibiotics (PEN, AMX, AMP, AZM, CTR, CX, GEN, OX, K). The phenotypic and genotypic characteristics of all the isolated *S. aureus* stains from milk of mastitis affected cow were shown in the Supplementary Table 1.

### 3.3 Molecular detection of MRSA

**Fig. 2:**
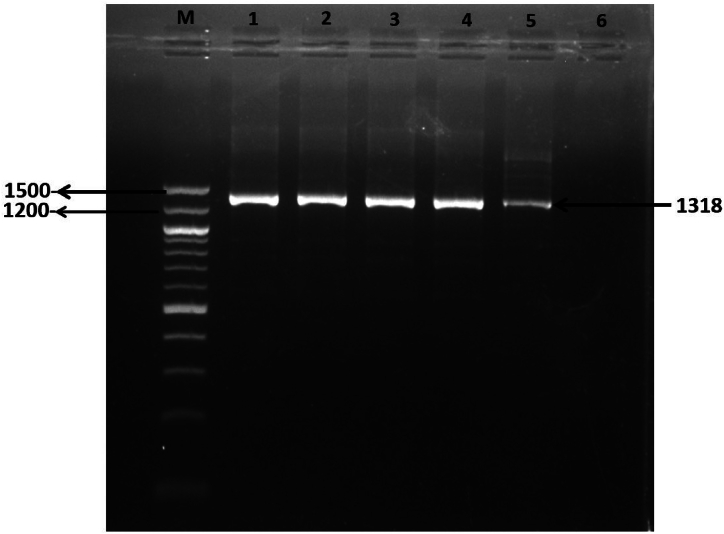
Gel electrophoresis of 23s *rRNA* gene of *S. aureus* showing an amplicon size of 1318bp; Lane M displayed a 100bp DNA ladder, while lane 1 represents positive sample, lanes 2 to 5 contained DNA from different samples and lane 6 represents negative control. Original and non-adjusted image is provided in the supplementary materials.

**Fig. 3:**
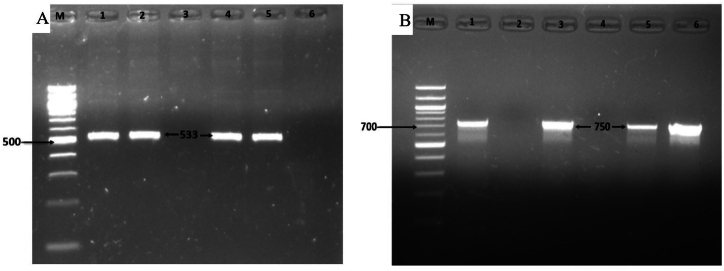
Virulence genes of *S. aureus*. (A) *mecA* gene of *S. aureus* showing an amplicon size of 533bp and (B) *coa* gene of *S. aureus* showing an amplicon size of 750bp; M = 100bp DNA ladder, 1–5 = different sample DNA and lane 6 represents negative control. Original and non-adjusted images are provided in the supplementary materials.

The presence of *S. aureus* was initially detected by amplifying the 23S rRNA gene (Fig. 2). Following this, PCR was utilized to confirm the prevalence of methicillin-resistant *S. aureus* (MRSA) among samples collected from clinical mastitis cases. Among the 56 *S. aureus* isolates analyzed, the methicillin resistance gene *mecA* was detected in 12 isolates, constituting 21.42 % of the total. (Fig. 3A). All the 12 isolates positive for *mecA* were phenotypically resistant to oxacillin and/or cefoxitin and MDR in nature. Among these 12 MRSA isolates, 4 found in MDR pattern 3, 4 found in MDR pattern 5 while the remaining 4 isolates were associated equally with patterns 6 and 7, respectively.

### 3.4 Detection of virulence genes

**Fig. 4:**
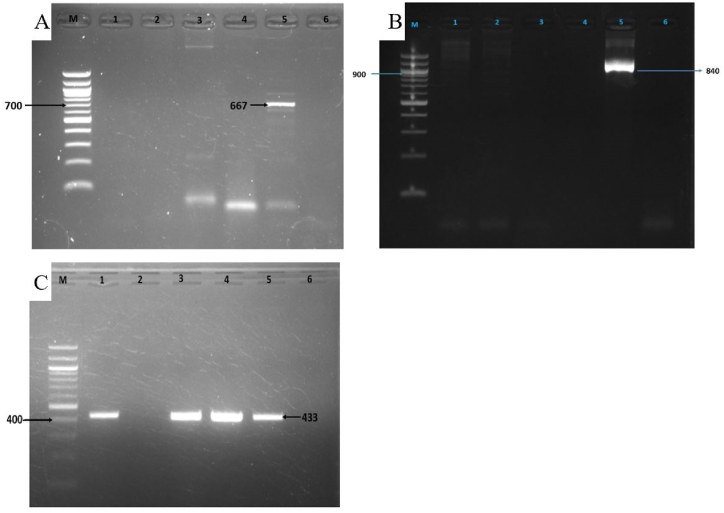
Virulence genes of *S. aureus*. (A) *seb*, (B) *hlb* and (C) *pvl* genes of *S. aureus* showing an amplicon size of 667bp, 840 and 433bp, respectively; M = 100bp DNA ladder, 1–5 = different sample DNA and lane 6 represents negative control. Original and non-adjusted images are provided in the supplementary materials.

Table 5 displays the occurrence of genes associated with virulence, including coagulase, enterotoxins, hemolysins, panton-valentine leucocidin, and genes involved in biofilm formation. This study found 50*S. aureus* isolates (89 %) as a coagulase positive (Fig. 3B). The enterotoxin *seb* gene was detected in 7.14 % (4/56) (Fig. 4A), however, none of the *S. aureus* showed positive for *sea* and sec genes. Moreover, it was found that 3.57 % and 10.71 % *S. aureus* isolated carried *hla* and *hlb* gene (Fig. 4B), respectively. *Pvl* gene was present in 17.85 % isolates (Fig. 4C). Gene responsible for biofilm formation (*bap*) was present in 64.28 % *S. aureus* isolates.

**Table 5:** Prevalence of virulence genes in isolated *S. aureus* isolates.

Factors	Gene	Number	Percentage
Coagulase	*Coa*	50	89.3
Enterotoxins	*sea*	0	0
	*seb*	4	7.14
	*sec*	0	0
Hemolysins	*hla*	2	3.57
	*hlb*	6	10.71
Panton-Valentine leukocidin	*pvl*	10	17.85
Biofilm	*bap*	36	64.28

### 3.5 Biofilm formation

**Fig. 5:**
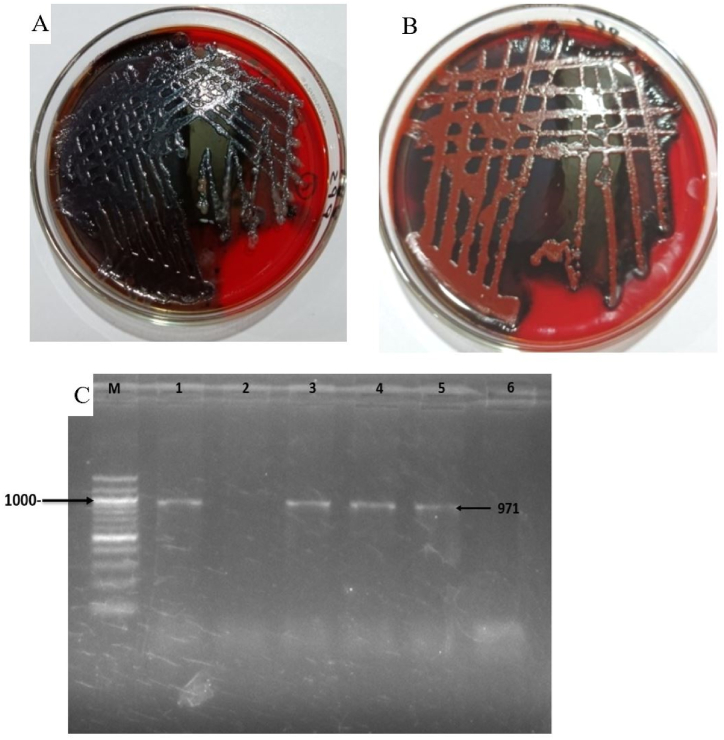
Biofilm formation characteristics of *S. aureus*; (A) Strong biofilm-producer in CRA media, (B) Moderate biofilm-producer in CRA media, (C) Gel electrophoresis of *bap* gene of *S. aureus* showing an amplicon size of 971bp; M = 100bp DNA ladder, 1–5 = different sample DNA and lane 6 represents negative control. Original and non-adjusted images are provided in the supplementary materials.

Presence of biofilm formation abilities in bacteria causing more resistant to antibiotics leading to development of MDR bacteria. Next, we determined the abilities of the MRSA isolates to develop biofilm by CRA method. Phenotypically, 42 strains (75 %) demonstrated the capacity to form biofilm in laboratory conditions (Fig. 5A and B). The findings regarding biofilm development indicated that every one of the 12 MRSA strains (100 %) exhibited the ability to form biofilm. In addition, the *bap* gene present in 36 isolates (64.28 %) and we observed the genotypic confirmation of biofilm formation gene (*bap* gene) in all MRSA isolates (Fig. 5C).

## 4 Discussion

Numerous bacteria are implicated in bovine mastitis, with *S. aureus* recognized as one of the foremost and widespread causal agents in Bangladesh and elsewhere [29,30]. A thorough understanding of mastitis's pathogen profile and its virulence characteristics is crucial for the effective control and management of this condition in dairy animals [31]. The prevalence and challenges associated with *S. aureus* infections in dairy cattle are widely recognized in South Asia, including Bangladesh, India, and Nepal [32]. The region shares common issues related to the management of bovine mastitis, a significant concern for dairy farmers due to its impact on milk production and animal health. Several studies across South Asia have reported a high prevalence of *S. aureus* in mastitis cases. The rise of MDR *S. aureus* strains is a growing concern across South Asia. The overuse and misuse of antibiotics in livestock, driven by the lack of strict regulatory controls and the easy availability of antibiotics, have contributed to the emergence of MDR strains [33]. This trend has been documented in various studies from India and Pakistan, where the prevalence of MDR *S. aureus* in dairy farms mirrors the findings in Bangladesh. The presence of these resistant strains complicates treatment efforts and leads to prolonged infections, higher treatment costs, and increased culling rates, all of which have significant economic implications for the dairy industry in the region. The present study reveals a prevalence of 46.66 % for *S. aureus* in clinical mastitis among bovines in the northern region of Bangladesh. This finding mirrors previous reports by Kasa et al. [34], Abebe et al. [35], and Abera et al. [36], who documented prevalence rates of 38.6 %, 51.2 %, and 46.7 %, respectively. However, Eid et al., [37]; Tesfaye et al. [38], Hoque et al. [18], and Tenhagen et al. [39] noted variations, reporting rates of 25.3 %,30.6 %, 72.7 %, and 5.7 %, respectively, for *S. aureus* in bovine mastitis cases. The prevalence of mastitis in cow varies due to different conditions such as management practices, pathogen prevalence, genetic factors, and environmental influences. Differences in diagnostic methods and study criteria also contribute to variation in reported prevalence rates. Factors like farm management practices, sample origins, and study methodologies further impact reporting consistency [18,35].

The utilization of antibiotics has long been a common practice in effectively treating bovine mastitis induced by *S. aureus*. β-Lactams, macrolides, and tetracyclines are commonly utilized in managing staphylococcal mastitis in cattle. Nonetheless, their efficacy is compromised by the rise of multidrug-resistant strains [5]. The outcome of treatment for infections caused by *S. aureus*, particularly when involving MDR strains or biofilm-forming phenotypes, is often less favorable and more complex compared to non-resistant or planktonic bacteria [[40], [41], [42]]. The presence of MDR strains significantly reduces the effectiveness of standard antibiotic therapies, leading to prolonged treatment durations, higher doses of antibiotics, and sometimes the necessity to use more potent or last-resort antibiotics, such as vancomycin. However, even these aggressive treatments may not guarantee success due to the high adaptability and resilience of MDR *S. aureus*. When *S. aureus* forms biofilms, the challenges associated with treatment are further amplified, as biofilms act as physical and chemical barriers, reducing antibiotic penetration and protecting the bacteria from the immune system and antimicrobial agents. This can result in chronic infections that persist despite ongoing treatment, particularly in veterinary settings like bovine mastitis, where biofilm-forming *S. aureus* strains can cause recurrent infections that are difficult to resolve. The combination of MDR and biofilm formation often leads to poor treatment outcomes, with higher rates of relapse, prolonged infections, and in some cases, the necessity to cull affected animals due to the inability to control the infection effectively [43,44]. The animals in this study were treated with antibiotics based on the antibiotic sensitivity results. It was observed that most of the animals took a longer time to recover from the infection (data not shown). After 5 weeks, all animals recovered except one, which subsequently developed gangrenous mastitis. These challenges underscore the need for alternative treatment strategies, such as biofilm-disrupting agents, combination therapies, and improved infection prevention practices, to enhance treatment efficacy and reduce the impact of these infections on animal health and productivity. In the present study, all isolated *S. aureus* strains underwent antimicrobial susceptibility testing. In this study, penicillin showed 100 % resistance to *S. aureus*, followed by amoxicillin, ampicillin and chlortetracycline. On the other hand, vancomycin, ciprofloxacin and gentamicin found most sensitive to the isolated *S. aureus*. Our results align with previous studies, which consistently document resistance to these antibiotics [19,29,45]. In our investigation, 36 out of 56 isolates were identified as multidrug-resistant (MDR). A significant contributing factor is the extensive and sometimes indiscriminate administration of antibiotics for mastitis treatment in dairy cattle. This selective pressure fosters the emergence of resistance genes in *S. aureus*, enabling their survival despite subsequent antibiotic interventions [46]. Additionally, *S. aureus* possesses a natural ability to acquire resistance determinants through horizontal gene transfer from other bacteria within the udder environment [47]. These factors combined contribute to the concerning rise of MDR-SA in milk from bovine mastitis. MDR *S. aureus* has been associated with increased morbidity and mortality, as well as longer hospital stays in human patients [48] and severe illness in animals [49]. Despite the challenges posed by MDR pathogens, recent studies have shown promising results in both in vitro and in vivo investigations of *S. aureus* infections, demonstrating effective antimicrobial strategies and the potential for successful treatment, providing hope for better control of diseases caused by these resistant strains [50].

The emergence of MRSA from the dairy source pose a threat to human health due to the possibilities of transferring from animals to human [51]. The presence of coagulase-positive *S. aureus* isolates that test negative for the *coa* gene is an important discrepancy. The coagulase enzyme is a key virulence factor, commonly associated with *S. aureus* infections, and the *coa* gene encodes the coagulase protein. However, several studies have reported instances where *S. aureus* isolates display coagulase activity but are negative for the *coa* gene [52]. These findings suggest possible genetic variations, mutations, or the existence of alternative genetic mechanisms responsible for coagulase production. MRSA isolates are also MDR which bear resistance genes on their chromosome cassette mec carries the *mecA* gene [53]. The presence of resistance genes such as *mecA* exacerbates the issue by reducing the efficacy of commonly used antibiotics like β-lactams, necessitating the use of more potent drugs, which may not always be available or may lead to adverse effects [54]. In this study, 22 out of 56 S aureus strains (39 %) exhibited phenotypic resistance to oxacillin. However, only 8 isolates (14 %) were genotypically confirmed to possess the methicillin resistance gene (*mecA*). This discrepancy underscores the complex nature of antibiotic resistance in *S. aureus*, where phenotypic resistance does not always correlate with the presence of known resistance genes like *mecA*. The variation observed may be attributed to several factors, including differential expression of the *mecA* gene, which can be influenced by regulatory elements and environmental conditions. Some *S. aureus* strains may carry alternative resistance mechanisms, such as mutations in other genes like *mecC* or modifications in penicillin-binding proteins (PBPs) that confer resistance to β-lactam antibiotics independently of *mecA* [55]. Additionally, phenotypic resistance could arise from non-genetic factors, such as the presence of biofilms or changes in cell wall structure, which can inhibit antibiotic efficacy even in the absence of *mecA*. This finding highlights the challenges in diagnosing and treating methicillin-resistant S aureus (MRSA) infections, as reliance solely on genotypic methods may overlook strains that display resistance through other pathways. Therefore, a comprehensive diagnostic approach that includes both phenotypic and genotypic assessments is essential for accurately identifying and managing MRSA infections, particularly in agricultural settings where varied environmental factors may influence resistance expression. Our results are consistent with the findings of Koupahi et al. [56], who reported a 47.72 % presence of the *mecA* gene in 220 *S. aureus* isolates. Similarly, Havaei et al. [57] observed a positive *mecA* test in 18.52 % of the samples, with 10 out of 54 *S. aureus* isolates carrying the gene. These studies further emphasize the need for a deeper understanding of the molecular mechanisms governing antibiotic resistance in *S. aureus* to improve treatment outcomes and resistance mitigation strategies.

This research has highlighted that all MRSA isolates demonstrated the ability to form biofilms in vitro, a trait that was genotypically confirmed by the presence of the *bap* gene. The *bap* gene plays a pivotal role in biofilm formation by encoding a protein that mediates cell-to-cell adhesion, which is essential for the initial stages of biofilm development. This adhesion is facilitated by the binding of the *bap* encoded protein to the bacterial cell surface, promoting the aggregation of cells into a biofilm matrix [58]. Remarkably, not all isolates capable of producing biofilms carried the *bap* gene, suggesting that alternative genetic pathways may also contribute to biofilm formation in these strains. This indicates the presence of other biofilm-associated genes or regulatory mechanisms that can compensate for the absence of the *bap* gene. For instance, other surface proteins or polysaccharide intercellular adhesins (PIAs) might be involved in promoting biofilm formation through different mechanisms, such as enhanced extracellular matrix production or increased cell surface hydrophobicity, which can also facilitate biofilm development [59]. These findings suggest that MRSA strains possess a diverse set of genetic tools to form biofilms, which could complicate treatment strategies and highlight the need for further research into the specific pathways involved.

This observation aligns with findings that propose various genes, including *icaA, icaD, icaC, fnbA, clfA, clfB*, and *fib*, may also exert significant influences on biofilm production [16]. Biofilm formation represents a crucial virulence trait for *S. aureus*, as it enhance the bacteria's resistance to antibiotics and its ability to elude the host's immune defenses [26].The discovery of multiple genes contributing to biofilm formation underscores the complexity of this process and highlights the potential for diverse strategies in managing MRSA infections. The *bap* gene encodes a surface protein that is crucial for biofilm development, significantly contributing to *S. aureus* ability to attach to host tissues and medical devices. This gene plays a key role in evading host immune responses and allowing the bacteria to persist within the host environment [58]. The presence of the *bap* gene in *S. aureus* isolates from mastitic milk samples suggests that these strains have the potential to form biofilms in the udder, which can lead to the persistence and chronicity of mastitis in animals. Moreover, biofilm formation by *S. aureus* has been associated with increased antimicrobial resistance, complicating treatment strategies and making infections more difficult to manage [60,61].

The study examined 56 *S. aureus* isolates, investigating 7 virulence genes to assess their potential pathogenicity. Results revealed a 17 % prevalence of the *pvl* gene among the *S. aureus* isolates. This gene, encoding the Panton-Valentine leukocidin toxin, induces leukocyte lysis, leading to tissue necrosis and inflammation. The detection of *pvl* gene in *S. aureus* isolates from milk underscores the virulence potential of these strains, suggesting their capacity to induce significant tissue damage and provoke inflammatory reactions [62]. The *seb* (staphylococcal enterotoxin B) gene encodes a heat-stable super antigen toxin that acts as a potent mitogen, triggering excessive activation of the host immune system and inducing inflammatory cytokine production. The presence of the *seb* gene in milk sample suggests the potential for these strains to elicit strong inflammatory responses in the udder tissues, contributing to the clinical manifestations of mastitis [63]. The *hla* (alpha-hemolysin) and *hlb* (beta-hemolysin) genes are responsible for producing toxins that form pores, disrupting membranes of host cells, which leads to cell lysis and tissue damage. The identification of these hemolysin genes in *S. aureus* isolates from mastitic milk samples highlights their potential for cytotoxicity and their capability to induce tissue destruction within the mammary gland. Additionally, alpha-hemolysin has been associated with evading host immune responses and establishing chronic infections, thereby contributing to the persistence of mastitis cases [64].

## 5 Conclusion

This study highlights the significant role of *S. aureus* as a leading cause of bovine mastitis in northern Bangladesh, reflecting global trends and its impact on veterinary and public health. The identification of *S. aureus* strains with high antibiotic resistance level including MRSA, raises concerns about the effectiveness of current treatment protocols for veterinary medicine and the potential threat to animal health and productivity. These isolates showed diverse resistance patterns, particularly to antibiotics commonly used in veterinary settings and poses virulence genes related to biofilm formation, adhesion, invasion and toxin production. The presence of MRSA in milk also indicates a potential risk to human health through food chain. These findings emphasize the urgent need for antimicrobial stewardship and surveillance in dairy farming to reduce the spread of antibiotic resistance. Continuous monitoring of antibiotic resistance patterns and virulence factors of *S. aureus* from animal is crucial for developing evidence-based strategies to manage and prevent mastitis, ensuring food safety and the health of animals and humans.

## CRediT authorship contribution statement

**Md. Mominul Islam:** Writing – review & editing, Writing – original draft, Validation, Investigation, Funding acquisition, Formal analysis, Conceptualization. **Md. Imran Hossain:** Writing – review & editing, Writing – original draft, Investigation, Formal analysis, Data curation. **Md. Sadequl Islam:** Writing – review & editing, Writing – original draft, Validation, Formal analysis. **Md. Golam Azam:** Writing – review & editing, Validation, Formal analysis. **Sajeda Sultana:** Writing – review & editing, Investigation, Data curation.

## Data and code availability

Data will be made available on request.

## Declaration of competing interest

The authors declare that they have no known competing financial interests or personal relationships that could have appeared to influence the work reported in this paper.
