# Supplementary material for: Prevalence, antibiotic resistance patterns, and virulence factors of Staphylococcus aureus isolates associated with bovine mastitis in northern Bangladesh
Source: Heliyon. 2025 Jan 21;11(3):e42107. doi: 10.1016/j.heliyon.2025.e42107 (PMC11830309; doi:10.1016/j.heliyon.2025.e42107)
Supplement: Multimedia component 1 [file mmc1.docx]

Supplementary table: Phenotypic and genotypic characteristics of *S. aureus* strains isolated from mastitic milk.

| SL no. | Strain number | Biofilm production | | MRSA | | Antibiotic resistance patterns | Virulence factors | | | | | | |
| --- | --- | --- | --- | --- | --- | --- | --- | --- | --- | --- | --- | --- | --- |
|  |  |  |  |  |  |  | *coa* | *sea* | *seb* | *sec* | *hla* | *hlb* | *pvl* |
|  |  | Phenotype | Genotype (*bap*) | Phenotype | Genotype  (*mec*A) |  |  |  |  |  |  |  |  |
| 1 | M03 | + | + | - | - | 2 | + | - | - | - | - | - | - |
| 2 | M04 | + | + | + | + | 5 | + | - | + | - | - | + | + |
| 3 | M07 | - | - | + | - | 7 | + | - | - | - | - | - | - |
| 4 | M11 | + | + | + | + | 6 | + | - | - | - | + | - | - |
| 5 | M12 | + | + | - | - | 8 | + | - | - | - | - | - | - |
| 6 | M13 | + | + | - | - | 8 | + | - | - | - | - | - | - |
| 7 | M16 | + | + | + | + | 3 | + | - | - | - | - | - | + |
| 8 | M18 | - | - | + | - | 7 | - | - | - | - | - | - | - |
| 9 | M19 | + | - | + | - | 5 | + | - | - | - | - | - | - |
| 10 | M21 | + | + | - | - | 8 | + | - | - | - | - | - | - |
| 11 | M24 | + | + | - | - | 2 | + | - | - | - | - | + | + |
| 12 | M25 | - | - | - | - | 8 | - | - | - | - | - | - | - |
| 13 | M30 | + | + | - | - | 8 | + | - | - | - | - | - | - |
| 14 | M31 | + | + | + | + | 3 | + | - | - | - | - | - | - |
| 15 | M32 | + | + | + | + | 5 | + | - | + | - | - | - | - |
| 16 | M34 | + | - | - | - | 8 | + | - | - | - | - | - | - |
| 17 | M36 | + | + | + | - | 1 | + | - | - | - | - | + | - |
| 18 | M38 | + | + | - | - | 2 | + | - | - | - | - | - | - |
| 19 | M39 | + | + | - | - | 7 | + | - | - | - | - | - | - |
| 20 | M40 | + | + | - | - | 8 | + | - | - | - | - | - | - |
| 21 | S02 | + | - | + | + | 7 | + | - | - | - | - | - | + |
| 22 | S04 | + | + | - | - | 8 | + | - | - | - | - | - | - |
| 23 | S05 | - | - | + | - | 7 | + | - | - | - | - | - | - |
| 24 | S06 | - | - | - | - | 4 | - | - | - | - | - | - | - |
| 25 | S09 | + | - | - | - | 8 | + | - | - | - | - | - | - |
| 26 | S10 | + | + | + | + | 6 | + | - | - | - | - | + | - |
| 27 | S13 | - | - | + | - | 5 | + | - | - | - | - | - | - |
| 28 | S17 | + | + | + | + | 5 | + | - | - | - | - | - | + |
| SL no. | Strain number | Biofilm production | | MRSA | | Antibiotic resistance patterns | Virulence factors | | | | | | |
|  |  |  |  |  |  |  | *coa* | *sea* | *seb* | *sec* | *hla* | *hlb* | *pvl* |
|  |  | Phenotype | Genotype (*bap*) | Phenotype | Genotype  (*mec*A) |  |  |  |  |  |  |  |  |
| 29 | S18 | + | + | - | - | 8 | + | - | - | - | - | - | - |
| 30 | S23 | + | - | + | - | 5 | - | - | - | - | - | - | + |
| 31 | S24 | + | + | - | - | 2 | + | - | - | - | - | - | - |
| 32 | S27 | + | + | + | - | 1 | + | - | - | - | - | + | - |
| 33 | S29 | + | + | + | + | 3 | + | - | + | - | - | - | - |
| 34 | S32 | - | - | - | - | 6 | + | - | - | - | - | - | - |
| 35 | S33 | + | + | - | - | 8 | + | - | - | - | - | - | - |
| 36 | S34 | + | + | + | - | 5 | + | - | + | - | - | - | - |
| 37 | S36 | + | + | - | - | 8 | + | - | - | - | - | - | - |
| 38 | S37 | + | - | - | - | 8 | + | - | - | - | - | - | - |
| 39 | S39 | + | + | + | + | 7 | + | - | - | - | - | - | + |
| 40 | A01 | - | - | - | - | 8 | + | - | - | - | - | - | - |
| 41 | A02 | + | + | - | - | 8 | + | - | - | - | - | - | - |
| 42 | A05 | - | - | + | - | 7 | - | - | - | - | - | - | - |
| 43 | A06 | + | + | - | - | 2 | + | - | - | - | - | - | - |
| 44 | A08 | + | + | - | - | 6 | + | - | - | - | - | - | - |
| 45 | A11 | - | - | - | - | 8 | + | - | - | - | - | - | + |
| 46 | A13 | + | + | - | - | 8 | + | - | - | - | - | - | - |
| 47 | A14 | + | + | + | + | 5 | + | - | - | - | - | + | - |
| 48 | A19 | + | + | - | - | 8 | + | - | - | - | - | - | - |
| 49 | A20 | + | + | - | - | 8 | + | - | - | - | - | - | - |
| 50 | A21 | - | - | + | - | 5 | + | - | - | - | - | - | - |
| 51 | A26 | - | - | - | - | 2 | - | - | - | - | - | - | - |
| 52 | A29 | + | + | - | - | 7 | + | - | - | - | - | - | + |
| 53 | A30 | + | + | + | + | 3 | + | - | - | - | - | - | + |
| 54 | A31 | + | + | - | - | 8 | + | - | - | - | - | - | - |
| 55 | A36 | + | + | - | - | 4 | + | - | - | - | - | - | - |
| 56 | A37 | + | + | - | - | 5 | + | - | - | - | - | - | - |

‘+’ denotes presence respective phenotype or gene; ‘-’ denotes absence respective phenotype or gene.
